# Good manufacturing practice production of CD34^+^ progenitor-derived NK cells for adoptive immunotherapy in acute myeloid leukemia

**DOI:** 10.1007/s00262-023-03492-6

**Published:** 2023-07-21

**Authors:** P. K. J. D. de Jonge, P. M. M. van Hauten, L. D. Janssen, A. L. de Goede, M. M. Berrien-Elliott, J. M. R. van der Meer, C. M. Mousset, M. W. H. Roeven, M. Foster, N. Blijlevens, W. Hobo, T. A. Fehniger, J. H. Jansen, N. P. M. Schaap, H. Dolstra

**Affiliations:** 1 Department of Laboratory Medicine, Laboratory of Hematology, Radboud University Medical Center, Geert Grooteplein 8, P.O. Box 9101, 6500 HB Nijmegen, The Netherlands; 2 Department of Hematology, Radboud University Medical Center, Nijmegen, The Netherlands; 3 Department of Pharmacy, Radboud University Medical Center, Nijmegen, The Netherlands; 4 Department of Medicine, Division of Oncology, Washington University School of Medicine, St Louis, MO USA

**Keywords:** Natural killer (NK) cells, Immunotherapy, Adoptive transfer, Good manufacturing practice (GMP), Cell manufacturing

## Abstract

Allogeneic natural killer (NK) cell-based immunotherapy is a promising, well-tolerated adjuvant therapeutic approach for acute myeloid leukemia (AML). For reproducible NK cell immunotherapy, a homogenous, pure and scalable NK cell product is preferred. Therefore, we developed a good manufacturing practice (GMP)-compliant, cytokine-based ex vivo manufacturing process for generating NK cells from CD34^+^ hematopoietic stem and progenitor cells (HSPC). This manufacturing process combines amongst others IL15 and IL12 and the aryl hydrocarbon receptor antagonist StemRegenin-1 (SR1) to generate a consistent and active NK cell product that fits the requirements for NK cell immunotherapy well. The cell culture protocol was first optimized to generate NK cells with required expansion and differentiation capacity in GMP-compliant closed system cell culture bags. In addition, phenotype, antitumor potency, proliferative and metabolic capacity were evaluated to characterize the HSPC-NK product. Subsequently, seven batches were manufactured for qualification of the process. All seven runs demonstrated consistent results for proliferation, differentiation and antitumor potency, and preliminary specifications for the investigational medicinal product for early clinical phase trials were set. This GMP-compliant manufacturing process for HSPC-NK cells (named RNK001 cells) is used to produce NK cell batches applied in the clinical trial ‘Infusion of ex vivo-generated allogeneic natural killer cells in combination with subcutaneous IL2 in patients with acute myeloid leukemia’ approved by the Dutch Ethics Committee (EudraCT 2019-001929-27).

**Supplementary Information:**

The online version contains supplementary material available at 10.1007/s00262-023-03492-6.

## Introduction

Acute myeloid leukemia (AML) is the most common type of acute leukemia in adults and incidence increases with age [1]. Treatment with intensive chemotherapy and/or hypomethylating agents (HMA) combined with venetoclax can result in complete remission (CR), often followed by consolidation with allogeneic stem cell transplantation (allo-SCT). Nevertheless, prognosis remains poor due to high relapse rates. Currently, 35–40% of younger and 5–15% of older AML patients are alive 5 years after diagnosis. Relapse occurs in 20–80% of patients, depending on the genomic risk classification and response to induction chemotherapy. Furthermore, persistent minimal residual disease (MRD) prior to allo-SCT has been associated with increased relapse rates and decreased survival [2]. Therefore, novel adjuvant therapies are needed to induce deeper remission before allo-SCT.

Allogeneic natural killer (NK) cell-based immunotherapy is a promising, non-toxic adjuvant therapeutic approach for AML. Various studies have shown that allogeneic NK cell infusion following lymphodepleting conditioning is well-tolerated and exerts anti-leukemia activity, achieving CR in up to 47% of the patients [3–7]. Generally, allogeneic NK cell products are enriched from peripheral blood (PB) of haplo-identical donors followed by overnight activation with Interleukin (IL)2, IL15 or the combination of IL12/IL15/IL18 [4, 5, 7–10]. However, because PB-NK cells have limited expansion capacity in vitro, have limited life spans in vivo, and represent a small fraction of peripheral white blood cells, obtaining sufficient cell numbers without T cell contamination is a major obstacle for adoptive transfer [3, 5, 11, 12]. Contaminating alloreactive T cells harbor the risk of graft-versus-host disease (GvHD) induction, especially when IL2 or IL15 is co-administered in vivo to boost NK cell survival, proliferation and activity. To increase NK cell numbers, so called feeder cell lines have been designed, like irradiated target cell line K562, genetically modified to express membrane-bound IL15 or IL21, resulting in > 10.000-fold expansion of PB-NK cells in 5 weeks [13, 14]. Although, the first clinical trials show safety, clinical efficacy can be further improved [15, 16]. In addition, PB-enriched cell products contain high donor-intervariability [3, 12].Therefore, preferable for adoptive immunotherapy is the development of homogeneous and scalable allogeneic NK cell products.

Autologous NK-cell products have shown not to be capable of achieving a potent anti-tumor response, possibly due to the interaction with MHC-class I molecules [17, 18]. Moreover, the expansion efficiency and functional status of autologous NK cells were limited when compared to allogeneic NK cells, as autologous cells were often obtained from heavily pretreated patients [12]. Next, NK cell lines such as NK-92 have been studied in several clinical trials. While large numbers could be generated and clinical responses were observed, there is a need for irradiation of the product to prevent uncontrolled proliferation after infusion. This limits the immunotherapeutic potential of cell lines as in vivo expansion is correlated to treatment outcome [5].

Alternatively, NK cells can be generated ex vivo from hematopoietic stem and progenitor cells (HSPC) or induced pluripotent stem cells (iPSC) [19]. Previously, we reported good manufacturing practice (GMP) manufacturing and clinical results from a phase I trial using NK cells derived from umbilical cord blood (UCB)-derived CD34^+^ HSPC using heparin-based culture media and amongst others IL2 and IL15 [20–23]. We demonstrated that infusion of up to 30 × 10^6^/kg of these cells is feasible, safe and well-tolerated in older AML patients, who were ineligible for allo-SCT [21]. Recently, we revised the culture protocol by including the aryl hydrocarbon receptor (AhR) antagonist StemRegenin-1 (SR1), which enhances expansion of CD34^+^ HSPCs and upregulates NK-cell-specific transcription factors, and combining IL15 with low-dose IL12, which resulted in more robust expansion, strong potency, and higher maturation potential in vivo compared to previous IL2 based methods [24, 25]. In addition, these SR1/IL15/IL12-induced HSPC-NK cells have shown high cytotoxicity against AML cell lines and primary AML cells in vitro [24, 26, 27]. Here, we present data of pre-clinical process development and the qualification of our GMP-compliant manufacturing process for SR1/IL15/IL12 induced HSPC-NK cells (named RNK001 cells). Next, we describe our phase I/IIa clinical trial protocol to investigate the safety and efficacy of RNK001 cells in patients with AML or myelodysplastic syndrome with excess blasts (MDS-EB2) with residual disease, with and without in vivo IL2 cytokine support (EudraCT 2019-001929-27).

## Materials and methods

### HSPC-NK cell generation

HSPC-NK cells were generated from UCB-derived CD34^+^ progenitor cells. UCB units were derived from the Radboudumc cord blood bank (banked after written informed consent) or collected at caesarean sections using CB-collect bags (Fresenius Kabi) after written informed consent (approved by the Radboudumc Committee for Medical Research Ethics CMO 2014/226). When units from the Radboudumc cord blood bank were used, cryopreserved UCB units were thawed and CD34^+^ cells were magnetically isolated using the CliniMACS plus (Miltenyi Biotec). When collected at caesarean sections, CD34^+^ cells were magnetically isolated and cryopreserved from the mononuclear cells using a CD34 microbead kit (cat# 130-046-702, Miltenyi Biotec) after Ficoll-Paque separation within 24 h.

**Fig. 1:**
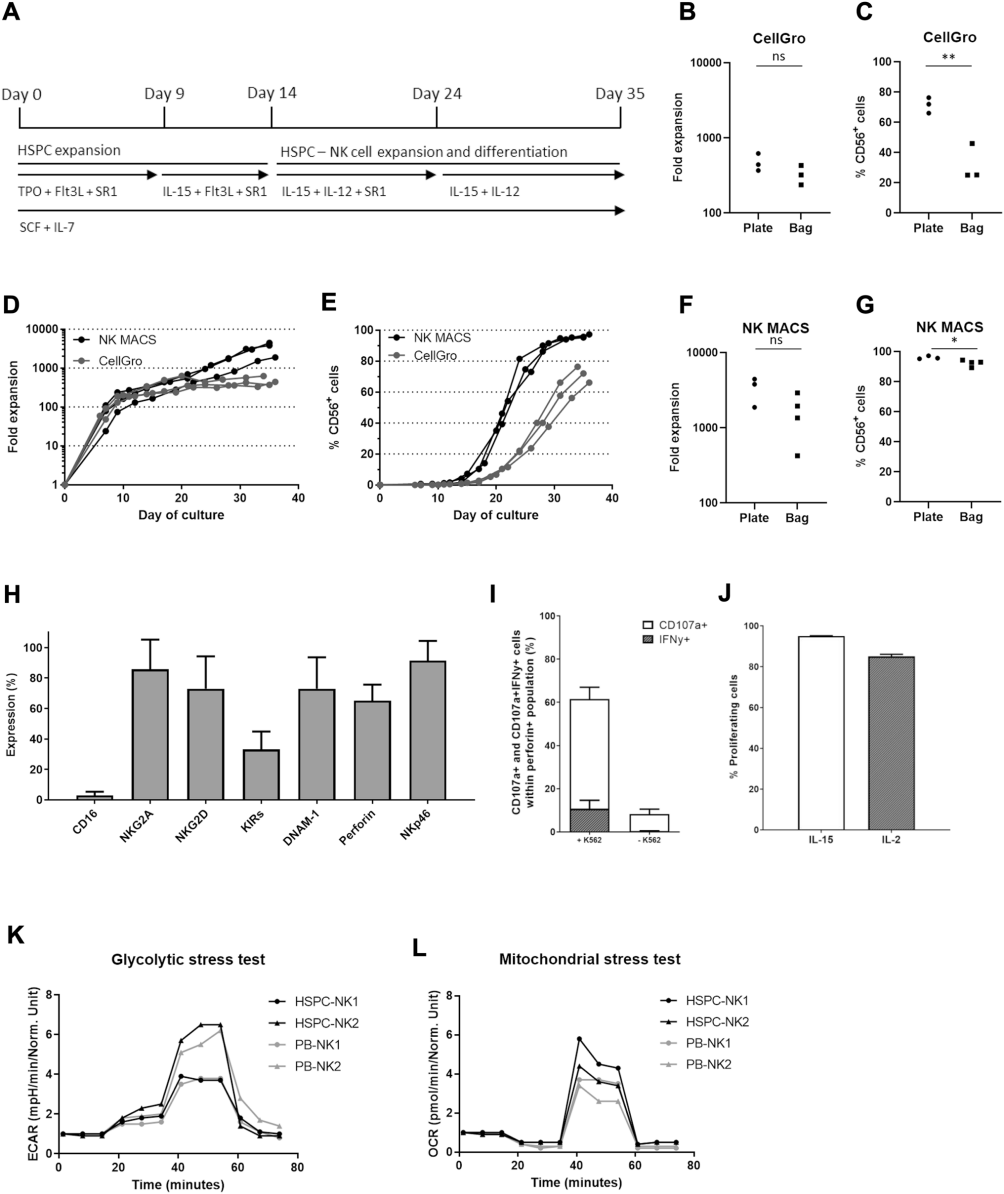
Translation of HSPC-NK cells with IL15/IL12/SR1-protocol from 6-well plate to a closed system cell culture bag using NK MACS medium. **A** Representation of the cytokine-based ex vivo culture protocol used for the generation of HSPC-NK cells. **B** Comparison of fold expansion of CD45^+^ expressing cells of the same donor measured at day 34–36, cultured in 6-well culture plates or cell culture bags using CellGro DC medium (n = 3). **C** Comparison of % CD45^+^CD56^+^ expressing cells of the same donor measured at day 34–36, cultured in 6-well plates or cell culture bags using CellGro DC medium (*p* = 0.007, n = 3). **D** Comparison fold expansion of CD45^+^ expressing cells during cell culture in CellGro DC medium or NK MACS medium, in 6-well culture plates (n = 3). **E** Comparison of % CD45^+^CD56^+^ expressing cells during cell culture in CellGro DC medium or NK MACS medium, in 6-well culture plates (n = 3). **F** Comparison of fold expansion of CD45^+^ expressing cells measured at day 35–36, cultured in 6-well culture plates or cell culture bags using NK MACS medium (n = 3–4). **G** Comparison of % CD45^+^CD56^+^ expressing cells of the same donor measured at day 35–36, cultured in 6-well plates or cell culture bags using NK MACS medium (*p* = 0.04, n = 3–4). **H** Mean expression of maturation and activation markers after 35 days, cultured in 6-well plates (n = 3, mean + SD). **I** Percentage of CD107a and IFNγ expressing CD56^+^Perforin^+^ NK cells, cultured in 6-well plates, with or without 4 h stimulation with K562 E:T ratio of 1.5:1 (n = 3, measured in singlet (unstimulated) or duplo (stimulated), mean + SEM). **J** Percentage proliferating NK cells, cultured in 6-well plates, after 5 days incubation with 1000 U/ml IL2 or 12,8 ng/ml IL15 (n = 2, measured in triplo, mean + SEM). Glycolytic (**K**) en mitochondrial (**L**) stress test of two HSPC-NK donors, each measured in triplo and two PB-NK donors, measured in duplo (PB-NK1) or triplo (PB-NK2). Values normalized to 1 at timepoint 0. Statistical analyses in **B**, **C**, **F** and **G** performed with two-tailed unpaired T-test

Isolated CD34^+^ cells were either cultured in CellGro Dendritic Cell (DC) specific medium (CellGenix) or NK MACS medium (Miltenyi Biotec) containing 2 or 10% Human serum (HS; Sanquin Bloodbank, Amsterdam, The Netherlands) and a specific mix of TPO (25 ng/ml, Immunotools, 500 U/ml, Miltenyi Biotec), FLT3L (25 ng/ml, Immunotools, 25 U/ml, Miltenyi Biotec), SCF (25 ng/ml, Immunotools, 25 U/ml, Miltenyi Biotec), IL7 (25 ng/ml, Immunotools, 2000 U/ml, Miltenyi Biotec), IL15 (50 ng/ml, Immunotools, 1000 U/ml, Miltenyi Biotec), IL12 (0.2 ng/ml, Immunotools, 0.25 U/ml, R&D systems) and/or SR1 (5 µM, Ardena) according to Fig. 1A (cytokines in ng/ml (Immunotools) for pre-validation and U/ml (Miltenyi Biotec and R&D systems) for GMP validation). 2% HS was used in differentiation medium for all cultures, except NK6 and NK7 in which 10% was used. Cells were cultured in 24 or 6-well plates (Corning) or VueLife 118/290/750 AC cell culture bags (Saint-Gobain). Cell concentration was maintained at around 2 million/ml by diluting the culture volume 1:3, 1:4 or 1:5 with fresh culture medium based on Trypan Blue Exclusion cell counting for both pre-validation and GMP validation. Medium volumes were based on calculated volumes per well/bag and was not measured. Cells were split when adding medium would result in > 5 ml in the 6-well culture or transferred from one 118 AC culture bag to two 750 AC culture bags at day 7 of the GMP validation. Cells were harvested and washed twice using CliniMACS PBS/EDTA buffer (Miltenyi Biotec) containing 0.5% Human Serum Albumin (HSA, Prothya Biosolutions). Finally, 1.0–3.0 × 10^9^ RNK001 cells were formulated in 500 ml 0.9% NaCl (Baxter) containing 5% HSA (Prothya Biosolutions). Validation runs NK1–NK7 were performed according to GMP-compliant manufacturing protocol.

### Tumor cell lines

K562 (RRID:CVCL_0004) were obtained from ATCC and cultured in Iscove’s Modified Dulbecco’s medium (IMDM; Gibco, #12440061) containing 10% FCS. Cell lines were cultured up to three months. Every 6 months, all cells in culture at that time were tested for mycoplasma with MycoAlertTM Mycoplasma Detection Kit (Lonza, #LT07-418).

### Purity and yield

For the CellGro vs NK MACS comparison, NK cell purity was determined by staining with CD45-ECD (Beckman Coulter, #A07784) and CD56-PECy7 (Biolegend, # 318318) at 4 °C for 30 min and 7-AAD (1:1000 diluted, Sigma, #A9400) was added before acquiring the cells on the FC500 flowcytometer (Beckman Coulter). Data was analyzed using Kaluza V2.1.3. For validation runs, NK cell purity and absolute cell count was performed by the Radboudumc Laboratory for Hematology Immunophenotyping unit (ISO15189 certified). Total nucleated cell yield and fold expansion was based on trypan blue exclusion counting before cell harvesting.

### Phenotyping

HSPC-NK cell phenotype was determined by staining for CD56-PE-Cy7 (Biolegend, #318318), NKG2A-APC (Beckman Coulter, #A60797), CD16-FITC (Biolegend, #302006), pan-KIRs-PE (Biolegend, #339506, 312606 and 312708), DNAM-1-FITC (BD, #559788), NKp46-PE (Biolegend, #331908) and NKG2D-APC (Biolegend, #320808) (CellGro vs NK MACS) or CD56-BV510 (Biolegend, #318340), CD45-BV421 (Biolegend, #368522), NKG2A-PE-Cy7 (Beckman Coulter, #B10246), DNAM-1-FITC (Biolegend, #337104), NKp46-PE (Biolegend, #311908), NKp44-PE (Biolegend, #325108), NKp30-APC (Biolegend, #325210) and NKG2D-APC (Biolegend, #320808) (GMP validations runs). Briefly, 200.000 cells were washed using PBS/0.5% BSA and incubated with antibodies in PBS/0.5% BSA at 4 °C for 30 min. Cells were then washed twice with PBS/0.5% BSA and resuspended in PBS/0.5% BSA containing Sytox Blue (1:5000 diluted, invitrogen, #S34857) for CellGro vs NK MACS experiments or 7-AAD (1:1000 diluted, Sigma, #A9400) for GMP validation runs. Cells were acquired on the Gallios (CellGro vs NK MACS) or Navios (GMP validation runs) flowcytometers and analyzed using Kaluza V2.1.3.

### RNK001 potency

RNK001 potency was determined by challenging them with K562 cells. Briefly, 150.000 RNK001 cells were co-cultured with 100.000 K562 cells in the presence of CD107a-PECy7 (Biolegend, #328618) at 37 °C for 4 h. After 1 h, Brefeldin A (BD Bioscience, #5550229) was added. Next, cells were harvested and stained with CD56-BV510 (Biolegend, # 318340) and eFluor780 (eBiosciences, #65-0865-14) at 4 °C for 30 min. Cells were then fixed and permeabilized using eBiosciences Fix/Perm buffer (#00-5123-43, #005223-56 and #00-8333-56). Finally, cells were stained intracellularly for IFN-γ-FITC (BD bioscience, #554700) and Perforin-PE (Biolegend, #308106), washed, acquired on the Gallios flowcytometer (Beckmann Coulter) and analyzed using Kaluza V2.1.3.

### Microbiology

RNK001 cells were tested according to European Pharmacopoeia (Ph. Eur.) 2.6.1 (sterility) and 2.6.14 (endotoxins) by Eurofins Bactimm. As the product is infused freshly, no sterility or endotoxin data is available at the time of infusion. Therefore, an in-process sterility and endotoxin test is performed at day 14/15 of the culture which is used for initial release. Pooled human serum is tested according to Ph. Eur. 2.6.7 (mycoplasma) by Eurfins MicroSafe Laboratories.

### NK cell proliferation assay

After RNK001 generation, cells were rested in NK MACS/2% HS + 5 ng/ml rhIL15 (Immunotools). Eight days after resting, NK cells were labeled with eFluor450 (eBioScience, #65-0842-85) and cultured in RPMI + 10% HS with 12.8 ng/ml rhIL15 or 1000 U/ml rhIL2 (Chiron Corporation) or without cytokines. Medium was refreshed on day 3 and analysis was performed on day 6. Dead cells were excluded using 7-AAD. Proliferation gate was set on 1% in the no cytokine control. NK cell numbers were based on CD56 gating events (CD56^−^ PE-Cy7, Beckman Coulter, #A21692) and measuring for a fixed time.

### Metabolic assays

The XF96 Extracellular Flux Analyzer (Seahorse, Agilent) was used to measure extracellular acidification rates (ECAR) and oxygen consumption rates (OCR). The Glycolytic stress (GST) tests were performed in DMEM (cat#D5030, Sigma) containing 143 mM NaCl (cat#3624-01, Baker) and 3 ml/l Phenol Red (cat#P0290, Sigma), supplemented with 2 mM l-glutamine (cat#35050-061, Life Technologies). For mitochondrial stress tests (MST), 25 mM d-glucose (cat#4912-12, VWR) and 1 mM sodium pyruvate (cat#360-070, Life Technologies) was added to the GST medium. GST was measured in response to 80 mM d-glucose, 18 µM oligomycin (cat#75351, Sigma) and 1 M 2-deoxy-d-glucose (2DG, cat#D6134, Sigma). MST was measured in response to 16 µM oligomycin, 4.5 µM fluoro-carbonyl cyanide phenylhydrazone (FCCP, cat#C2920, Sigma), 10 µM rotenone (cat#R8875, Sigma) and 10 µM antimycin A (cat#A8674, Sigma). PB-NK control cells were isolated from a buffy coat (Sanquin Blood Bank, Nijmegen, The Netherlands) using a CD56 microbead kit (cat# 130-050-401, Miltenyi Biotec) after Ficoll-Paque separation. Isolated CD56^+^ cells were rested for 7 days in RPMI-1640 containing 10% HS and 1 ng/ml IL15 (Immunotools). Half of the medium was replaced every other day.

### Mass cytometry

HSPC-NK SR1/15.12 (according to Fig. 1A) and HSPC-NK Hep/15.2 (according to [21]) differentiated cells were thawed and stained as previously described with the NK panel (panel according to [7, 8]). Briefly, dead cells were stained using cisplatin, then cell surface antigens were stained. Thereafter cells were fixed and permeabilized using eBiosciences Fix/Perm buffer, according to manufacturer’s instructions. Intracellular markers were stained in eBiosciences Perm Wash buffer. After staining, cells were then barcoded using Fluidigm 20-plex Pd Barcoding kit, according to manufacturer’s instructions. Cells were then collected on a Helios mass cytometer (Fluidigm) and analyzed using Cytobank ([28]). Live Cells were debarcoded ([29]), and CD45^+^CD19^−^CD14^−^CD3^−^CD56^+^NKp46^+^ cells identified by traditional gating. NKp46^+^ NK Hep/15.2, and NK SR1/15.12 cells were assessed using viSNE ([30]). Subsets within tSNE1/2 were identified using FlowSOM (clustering: tSNE1/2, 5 metaclusters, 25 clusters; [31]).

### Statistics

Statistical analysis was performed using Graphpad Prism software version 9.4.0. Two-tailed unpaired T-test, two-tailed Mann–Whitney test, Wilcoxon matched-pairs signed rank test, or two-way ANOVA were used as indicated in the figure legends. Significance was defined as *p* < 0.05 (*), *p* < 0.01 (**), *p* < 0.001 (***) and *p* < 0.0001 (****).

## Results

### HSPC-NK cell generation using NK MACS medium results in high proliferation and differentiation

We established a culture method to generate CD34^+^ progenitor-derived NK cells in the presence of the AhR antagonist SR1, IL15 and IL12 (Fig. 1A, [26]). To optimize the manufacturing process we changed the open 6-well culture system to a closed system using bags. Cell culture using CellGro DC medium in culture bags resulted in comparable proliferation compared to culture plates [median fold expansion in plates 443 times vs 322 times in bags (range 369–624 vs 237–431), non-significant (ns)]. However, it significantly delayed differentiation into CD56^+^ cells (median in plates at day 35 72% vs 25% in bags, range 66–76% vs 25–46%, *p* = 0.007) (Fig. 1B, C). Therefore, further optimization of the culture protocol was initiated by exploring HSPC-NK cell culture in 6-well plates for 35 days in NK specific NK MACS medium. This resulted in significantly increased proliferation (median fold expansion in CellGro DC medium 443 times vs 3810 times in NK MACS medium, range 369–624 vs 1879–4450, *p* = 0.02), as well as differentiation (median CD56^+^ NK cells in CellGro DC medium 72% vs 96% in NK MACS medium, range 66–76% vs 95–97%, *p* = 0.001) (Fig. 1D–E). Subsequently, these results were validated in closed system culture bags. Although cell expansion was lower in culture bags compared to 6-well plates (median fold increase in plates 3810 times vs 1654 times in bags, range 1879–4450 vs 426–2918, Fig. 1F, ns), final yield was 5 times higher for NK MACS medium compared to CellGro DC medium. This ensures a minimal desired dose of at least 1.0 × 10^9^ CD56^+^ NK cells for our phase I/IIa trial (EudraCT 2019-001929-27). A minimum of 1.0 × 10^9^ translates to ≥ 10 × 10^6^ NK cells/kg for patients up to 100 kg body weight, which has shown to be both safe and sufficient to accomplish a clinical effect [3, 5, 21]. Importantly, cell differentiation was well above 70% CD56^+^CD3^−^ expression (range 95–97% in plates vs 89–94% in bags, *p* = 0.04, Fig. 1G). This was a significant improvement compared to CellGro DC medium in culture bags (*p* < 0.001), where an average of only 32% could be achieved. These data demonstrate that high numbers of CD56^+^CD3^−^ HSPC-NK cells can be generated with our optimized SR1/IL15/IL12 protocol using NK MACS medium and cell culture bags.

### HSPC-NK cell generation using NK MACS medium results in functional NK cells with an active phenotype

To evaluate the functionality of the HSPC-NK cells generated using the NK MACS based protocol, we determined phenotype, potency, proliferation capacity and metabolic activity for independent cultures in 6-well plates. At the end of the culture the HSPC-NK cells expressed high levels of NKG2A, NKG2D, DNAM-1 and NKp46 (mean all ≥ 70%, mean ± SD: CD16 3.0% ± 2.4, NKG2A 85.8% ± 19.5, NKG2D 73.0% ± 21.3, KIRs 33.1% ± 11.8, DNAM-1 73.0% ± 20.6, Perforin 65.2% ± 10.5, NKp46 91.5% ± 12.9) (Fig. 1H). Moreover, functionality was evaluated after K562 challenge. 61.6% ± 4.5 of the CD56^+^perforin^+^ NK cells expressed degranulation marker CD107a and 10.7% ± 3.9 simultaneously produced IFNγ, which is comparable with NK cells generated in CellGro DC medium (Fig. 1I and Suppl Fig. 1, versus 8.3 ± 2.5 and 0.3 ± 0.2 respectively for unstimulated CD56^+^perforin^+^ NK cells, mean ± SEM) [26]. In addition, high proliferative capacity of 85.1% ± 1.0 for IL2 and 95.1% ± 0.1 for IL15 activated NK cells was shown (Fig. 1J). Finally, HSPC-NK cells were evaluated in glycolytic and mitochondrial stress tests, in comparison to PB-NK cells (Fig. 1K–L). Next, fold increase of the two donors per NK cell source, measured in duplo or triplo, were pooled. The changes in ECAR and OCR were analyzed showing comparable glycolysis [HSPC-NK 1.4 ± 0.4 fold change (mean ± SEM) vs PB-NK 0.8 ± 0.1], glycolytic capacity (HSPC-NK 4.3 ± 1.4 fold change vs PB-NK 3.9 ± 0.9), and maximal respiration (HSPC-NK 4.7 ± 0.7 fold change vs PB-NK 3.3 ± 0.2). All together these data show that the NK MACS based protocol results in robust proliferation and differentiation towards potent, functional and metabolically active CD56^+^ NK cells.

### Successful validation of the RNK001 manufacturing process

**Fig. 2:**
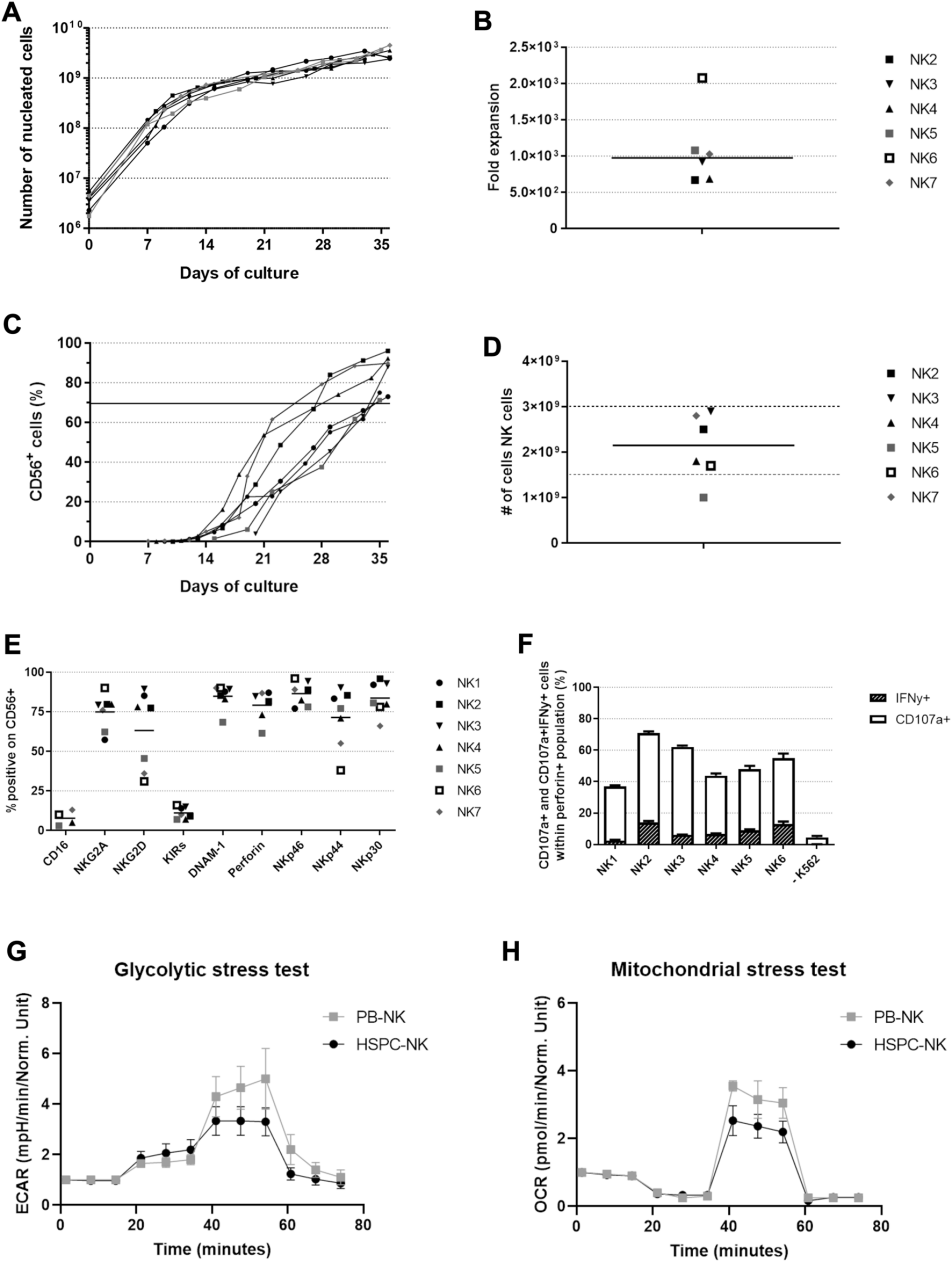
GMP grade production was successfully validated over seven production runs. Due to technical difficulties in harvesting the cells, no expansion data of the first donor is available. Expansion and differentiation was followed throughout the 35/36 day culture process and phenotype and functionality were determined at the end of culture. **A** Total number of nucleated cells (NC) during culture (n = 6). **B** Fold expansion of total nucleated cells (CD45^+^) at day 35/36 of the culture. Line at median (n = 6). **C** Development of CD45^+^CD56^+^ cells during the culture, indicating differentiation towards NK cells. All donors reached CD56 expression of > 70% (n = 7). **D** Absolute number of NK cells after harvesting and washing. Line at median (n = 6). **E** Expression of several activating and inhibitory receptors on CD45^+^CD56^+^ cells at day 35/36 of the culture (n = 4–7). **F** Percentage of CD107a and IFN-γ expressing CD56^+^perforin^+^ NK cells with and without 4 h stimulation with K562 E:T ratio of 1.5:1 (n = 6), measured in singlet (unstimulated) or duplo (stimulated). All bars represent the mean + SD. Glycolytic (**G**) and mitochondrial (**H**) stress test of three HSPC-NK donors, each measured in sixplo and two PB-NK donors, measured in duplo and triplo (mean ± SEM). Values normalized to 1 at timepoint 0

For validation of the manufacturing process seven independent runs were performed, using different UCB units, to establish the reproducibility of the culture process as documented in our investigational medicinal product dossier (Table 1). All cultures showed similar expansion patterns (Fig. 2A). Total number of nucleated cells ranged between 2.4 × 10^9^ and 4.5 × 10^9^ cells (670–2076 fold expansion) before harvesting the cells (Fig. 2A, B). The percentage of CD45^+^CD56^+^CD3^−^ cells ranged between 70 and 96%, with a mean of 84%, resulting in an absolute NK cell number of 1.0 × 10^9^ to 2.9 × 10^9^, with a mean of 2.1 × 10^9^, after harvesting and washing (Fig. 2C, D, Table 1). CD45^+^CD56^−^ cells consisted mainly of CD14^+^ and/or CD15^+^ cells (Supplementary Table 1). CD3^+^ T cell content and CD19^+^ B cell content was < 1.0 × 10^5^/kg and < 3.0 × 10^5^/kg, respectively (Table 1). Maturation of the NK cell product was established with mean ± SD expression for CD16 8% ± 5, NKG2A 75% ± 11, NKG2D 63% ± 25, KIRs 11% ± 4, DNAM-1 85% ± 8, perforin 79% ± 10, NKp46 87% ± 8, NKp44 71% ± 19 and NKp30 84% ± 11 (Fig. 2E, Table 1)*.* Moreover, functional activity after K562 challenging resulted in a mean CD107a^+^ expression of 52.7% ± 5.1 and IFNγ^+^ of 8.6% ± 1.8, which was comparable to culture in 6-well plates (Fig. 2F and Suppl Fig. 1, mean ± SEM, versus 4.5 ± 0.9 and 0.1 ± 0.0 respectively for unstimulated CD56^+^perforin^+^ NK cells (mean ± SD). Finally, HSPC-NK cells were evaluated in glycolytic and mitochondrial stress tests and compared to PB-NK cells. The changes in ECAR and OCR were analyzed showing adequate glycolysis [HSPC-NK 1.4 ± 0.5 fold change (mean ± SEM, n = 3) vs PB-NK 0.8 ± 0.1 (n = 2)] glycolytic capacity (HSPC-NK 2.5 ± 0.5 fold change vs PB-NK 3.9 ± 0.9), and maximal respiration (HSPC-NK 2.4 ± 0.4 pmol/min/normalized unit vs PB-NK 3.3 ± 0.2) (Fig. 2G, H). All cultures were sterile according to Ph. Eur. 2.6.1 and had low (< 0.3 EU/ml) endotoxin burden according to Ph. Eur. 2.6.14.

**Table 1:** Overview of critical QC results for the validation runs

	Start	Purity and viability						
	# CD34^+^ cells (× 10^6^)	# CD45^+^ CD56^+^ CD3- cells (× 10^9^)	Purity (%)	Viability (%)	T cell content (× 10^5^/kg)	B cell content (× 10^5^/kg)		
Target	≥ 1.0	≥ 1.0	≥ 70	≥ 70	< 1.0	< 3.0		
NK1	5.3	NA^#^	75	95	0.2	0.2		
NK2	4.1	2.5	92	99	0.2	0.1		
NK3	4.0	2.9	96	100	0.3	0.2		
NK4	3.5	1.8	88	99	0.1	0.1		
NK5	2.1	1.0	73	99	0.1	0.0		
NK6*	1.8	1.7	70	99	0.2	0.2		
NK7*	4.3	2.8	92	100	0.1	0.2		

	Phenotype of CD45^+^CD56^+^CD3^−^ cells (%)						Microbiology	
	NKG2A (%)	DNAM-1 (%)	NKp46 (%)	NKp44 (%)	NKp30 (%)	NKG2D (%)	Sterility	Endotoxin (EU/ml)
Target	> 30	> 30	> 30	> 30	> 30	> 30	Sterile	< 1.2
NK1	58	85	74	81	96	89	Sterile	< 0.3
NK2	80	86	89	85	96	77	Sterile	< 0.3
NK3	79	89	94	90	93	89	Sterile	< 0.3
NK4	80	83	82	71	80	78	Sterile	< 0.3
NK5	62	68	78	77	81	46	Sterile	< 0.3
NK6*	90	90	96	38	78	31	Sterile	< 0.3
NK7*	76	90	89	55	66	36	Sterile	< 0.3

T and B cell content were calculated based on a patient body weight of 70 kg

*Differentiation medium for NK6 and NK7 contained 10% human serum instead of 2% used in NK1-5

^#^Due to technical problems with cell harvesting, no reliable cell count could be obtained. This was solved for subsequent runs

**Fig. 3:**
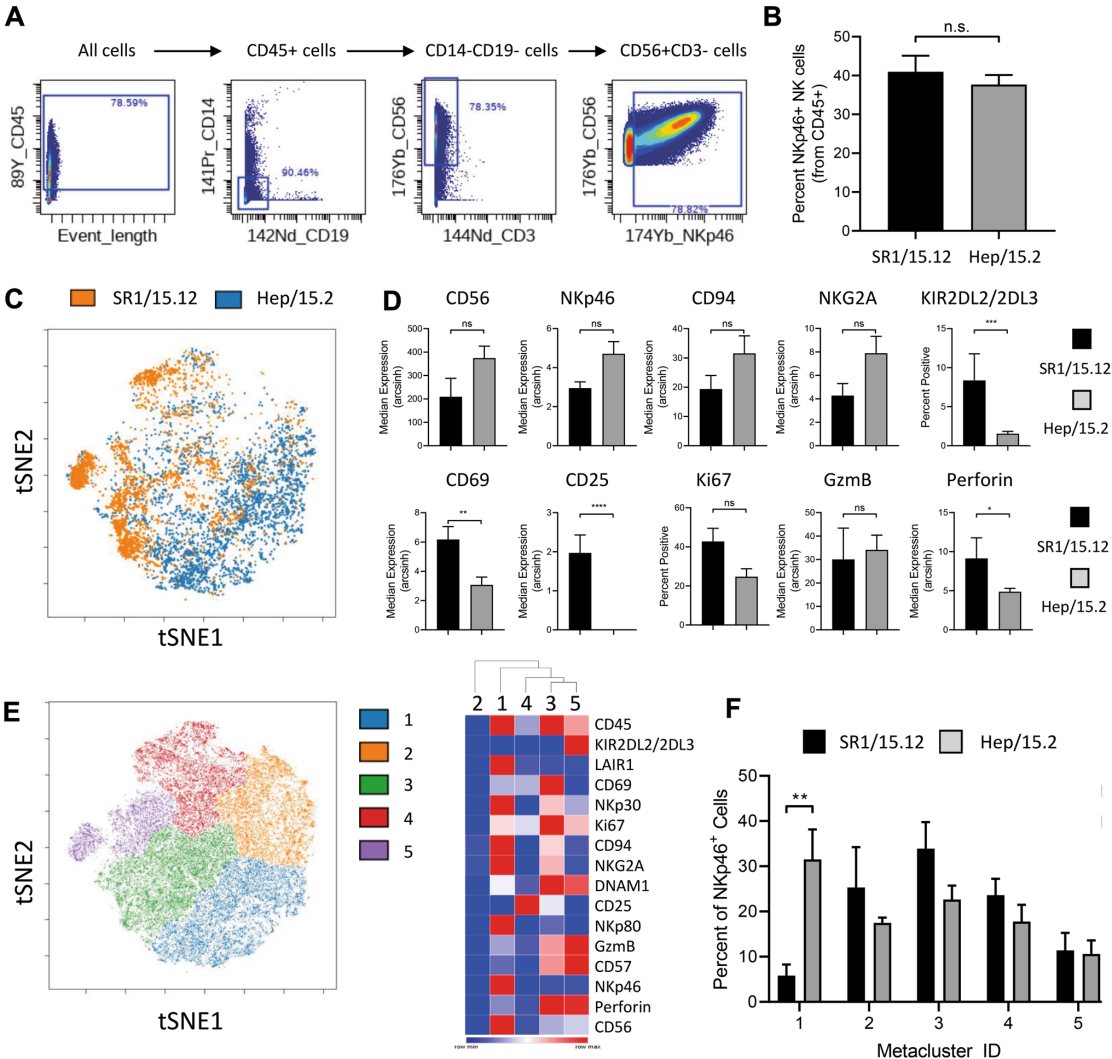
Mass cytometry of NK SR1/15.12 and NK Hep/15.2 showed that the improved SR1/15.12 resulted in a more mature and activated phenotype compared to the Hep/15.2 product. **A** Gating strategy used. **B** NKp46 expression within the CD56^+^CD3^−^ population was similar between culture methods. Both culture methods resulted in distinct phenotypes as visualized in by cluster analysis (**C**, **E**, **F**). **D** Positive population or Median expression of different markers, measured on CD45^+^CD14^−^CD19^−^ cells. Statistical analyses in B performed with two-tailed Mann–Whitney test, in D for CD56, NKp46, CD94, NKG2A, CD69 and CD25 a two-tailed unpaired T test and for KIR2DL2/2DL3, Ki67, granzyme B and Perforin a two-tailed Mann–Whitney test, in F a two-way ANOVA was used

Mass cytometry analysis was performed to compare the immunophenotype of HSPC-NK cells generated with the GMP-compliant SR1/IL15/IL12 manufacturing protocol and the ‘old’ Hep/IL15/IL2 culture process (Fig. 3). For this we compared the phenotype of NKp46^+^CD56^+^CD3^−^ NK cells (Fig. 3A, B). To complement Boolean gating, we also used an unbiased analysis approach (t-distributed stochastic neighbor embedding (tSNE)) and FlowSOM, a visualization technique to analyze high-dimensional data using self-organizing maps. Both culture methods resulted in distinct phenotypes of the generated NKp46^+^CD56^+^CD3^−^ NK cells as visualized by tSNE analysis (Fig. 3C). SR1/IL15/IL12 HSPC-NK cells were phenotypically more mature (increased KIR and perforin expression) and activated (increased CD69 and CD25 expression) compared to Hep/IL15/IL2 differentiated HSPC-NK cells (Fig. 3D). FlowSom analysis revealed five population clusters of which cluster 1 is significantly lower in SR1/IL15/IL12 HSPC-NK cells compared to Hep/IL15/IL2 differentiated HSPC-NK cells (Fig. 3E, F). This cluster 1 resembles an immature NK cell phenotype with high expression of CD56, CD94, NKG2A and NKp46, but low expression of perforin, GzmB, DNAM-1 and KIR. Other NK cell clusters identified were not significantly different between the two methods. Collectively, these data show that we established an improved GMP-compliant HSPC-NK cell generation process with potent proliferation, maturation and functional activity.

## Discussion

Here, we report on the development of a GMP-compliant manufacturing process of a CD34^+^ HSPC-derived NK cell product, named RNK001 cells. Highly functional allogeneic NK cells are generated using our SR1/IL15/IL12 culture protocol and a clinically relevant dose and purity (≥ 1.0 × 10^9^ CD56^+^ cells and ≥ 70% purity) could be achieved. Importantly, these NK cells exhibit a mature and functional phenotype, with high expression of activating receptors NKG2D, DNAM-1 and NCR, resulting in adequate degranulation, cytokine production and proliferation. The robustness of this culture protocol was confirmed and documented in an IMPD.

With this SR1/IL15/IL12 protocol, we generate large numbers of highly functional NK cells from CD34^+^ HSPCs derived from UCB units. While other HSPC sources (e.g. mobilized peripheral blood) could be used, UCB-derived HSPC generally result in higher NK cell yields that can be multiple folds greater (data not shown). Alternatively, HSPC-NK cells can be generated from human induced pluripotent stem cells (iPSCs) [32–34]. iPSC-derived NK cells derive from a clonal starting cell population resulting in an unlimited number of uniform mature NK cells. However, the strength of our product is the heterogeneity between the final NK cell products due to subtle differences in donors [21, 33, 34]. In addition, in our previous clinical trial in vivo HSPC-NK cell maturation was observed, indicated by the rapid acquisition of CD16 and KIR expression, while expression of most activating receptors was sustained [21, 25]. Importantly, no genetic mutation based on karyotyping was observed previously even after 42 days of culture [22].

**Fig. 4:**
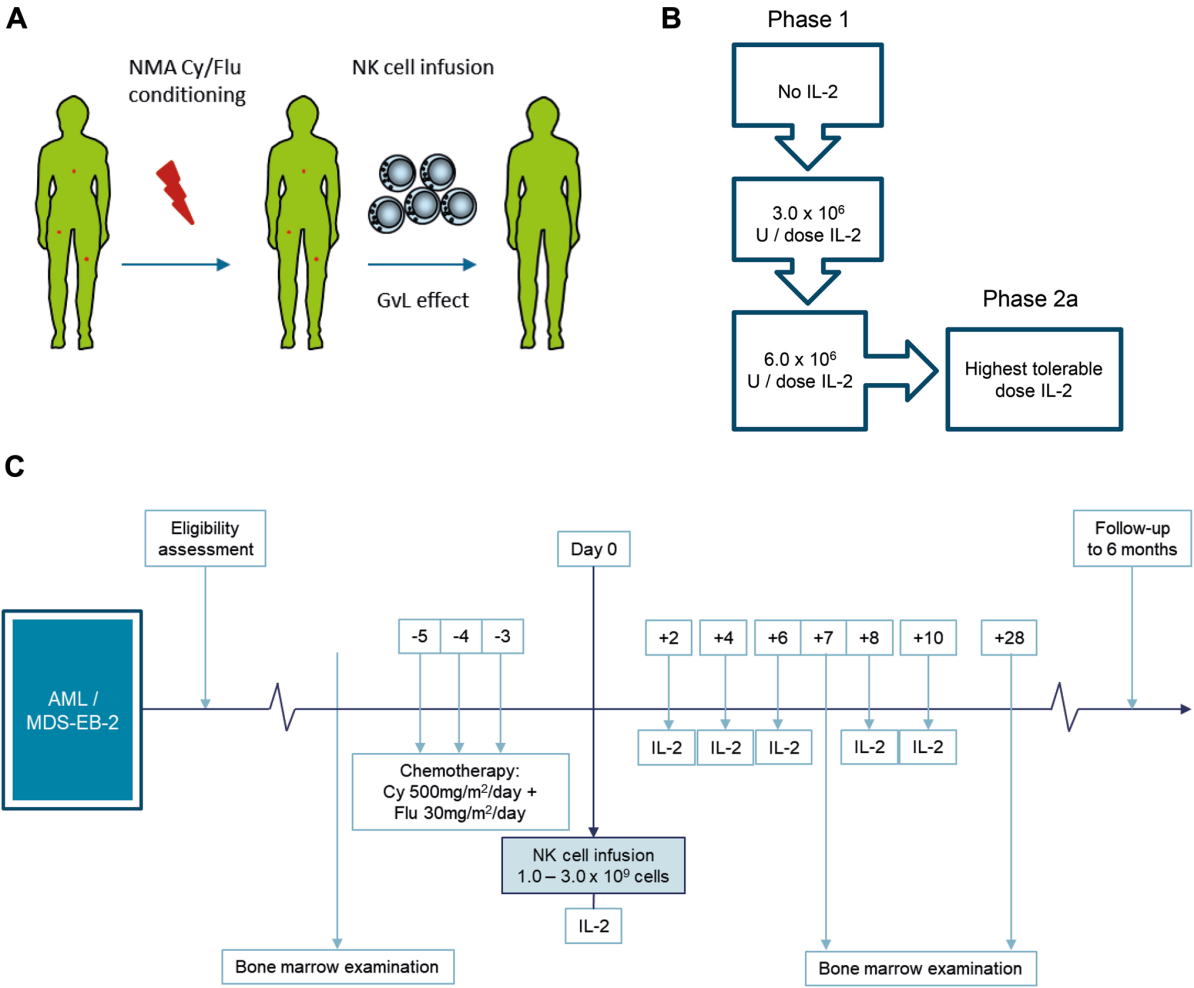
Clinical trial study design with RNK001 in AML/MDS-EB2 patients. **A** This study focusses on patients with either AML or MDS-EB2 de novo or relapsed or refractory disease. Every patient receives a non-myeloablative immunosuppressive conditioning regimen consisting of Cy/Flu, followed by a single infusion of allogeneic NK cells, hypothetically inducing a graft-versus-leukemia (GvL) effect. **B** The study is divided in two phases. Phase I is designed as a standard 3 + 3 dose escalating study, with increasing dose of IL2. Phase 2a is designed as a Simon’s optimal two stage single arm study. **C** Schematic overview of the study design. Patients with AML or MDS-EB2 will be screened on eligibility criteria. Five weeks before planned infusion generation of the HSPC-NK cells starts. Close to admission bone marrow investigation will be performed to establish disease status. Five days before infusion patients will receive IV NMA immunosuppression consisting of cyclophosphamide (500 mg/m^2^/day) and fludarabine (30 mg/m^2^/day) on days -5, -4 and -3. This Cy/Flu regimen will be administered in an inpatient hospitalized setting. On day 0 patients receive a fixed dose of 1.0–3.0 × 10^6^ NK cells. IL2 will be administered for six doses in total, given every other day, starting 4 h after NK cell infusion. Patients will be admitted to the hospital at least until the last IL2 administration. At day 7 bone marrow examination will be performed to evaluate NK cell homing to the bone marrow. At day 28 bone marrow examination will be performed to evaluate effect on disease status. Follow-up lasts 6 months

Allogeneic NK cell immunotherapy in general is known to be a safe and well-tolerated adjuvant therapy for patients with AML or MDS-EB2 [3, 5–9, 21, 35]. Prognosis of this patient category remains poor due to high relapse rates which is correlated with persistent MRD prior to allo-SCT. Therefore, we designed a prospective phase I/IIa trial called ‘Infusion of ex vivo*-*generated allogeneic NK cells in combination with subcutaneous (sc) IL2 in patients with acute myeloid leukemia’ (NCT04347616) to study the safety and efficacy of RNK001 cells with and without IL2 administration. The primary objective of phase I of the study is to evaluate the safety and toxicity of the infusion of RNK001 cells, with and without sc IL2, following a non-myeloablative immunosuppressive conditioning regimen. The primary objective of phase IIa of the study is to evaluate the effect of this immunotherapy regimen on disease activity in patients with AML or MDS-EB2, determined at day +28 post infusion (Fig. 4A, C).

Patients with AML (de novo and secondary) or MDS-EB2, aged ≥ 18 years, who have stable or at least non-rapidly progressive disease, with or without disease controlling medication and ineligible for allo-SCT will be included. Patients can be newly diagnosed, untreated, or suffer from relapsed or refractory disease after treatment. The first phase of the study is designed as standard 3 + 3 dose escalating study, comprising twelve patients. The second phase of the study is designed as Simon’s optimal two-stage single arm study, comprising seventeen patients. Prior to RNK001 infusion, patients will receive cyclophosphamide and fludarabine (Cy/Flu, cyclophosphamide 500 mg/m^2^/day and fludarabine 30 mg/m^2^/day, for 3 consecutive days) lymphodepleting chemotherapy. Then, patients will receive a single dose of 1.0–3.0 × 10^9^ RNK001 cells. In phase I patients will receive RNK001 cells without sc IL2, with 3.0 × 10^6^ units sc IL2 or with 6.0 × 10^6^ units sc IL2 (Fig. 4B). Patients will be screened for (serious) adverse events ((S)AEs) and GvHD, defining dose-limiting toxicities (DLTs). After establishing safety, phase IIa of the study will use the highest tolerable IL2 dose. Patients will be monitored for clinical toxicity, biological parameters in PB and BM and disease activity. Follow-up will last 6 months (Fig. 4C).

So far in other clinical studies up to 3.0 × 10^7^/kg peripheral blood or UCB-derived allogeneic NK cells have been administered to relapsed or refractory AML patients [3, 5, 21]. Allogeneic NK cell infusions were well-tolerated without cytokine release syndrome (CRS) or GvHD. Subcutaneous IL2 administration up to 9.0 × 10^6^ units was well-tolerated in earlier studies and IL2 side effects were dose related [3–5, 8, 36]. Common side effects include constitutional symptoms, hematologic toxicity and injection site reactions. Current therapies result in only 20% complete response in refractory or relapsed AML/MDS-EB2 patients. As allogeneic NK cell infusion has exerted promising anti-leukemia activity in the past, achieving up to 47% CR in poor prognosis AML patients with refractory or relapsed disease, our protocol could be a potential treatment option. This clinical trial protocol was approved by the Dutch Ethics Committee (Central Committee on Research Involving Human Subjects, CCMO) and the Competent Authority (Ministry of Health, Welfare and Sport, VWS) and is currently open for inclusion. The goal of our RNK001 platform in the future is (1) to induce deeper remissions before allo-SCT, (2) to boost the graft-versus-leukemia effect shortly after transplantation during the immunosuppressive window, (3) to treat an early relapse, or (4) as adjuvant therapy for allo-SCT ineligible patients.

Enhancing expansion and persistence of adoptive NK cell therapy will likely improve efficacy. In the previous PLMA25 study without exogenous cytokines, chimerism data showed transient HSPC-NK cell persistence in peripheral blood up to 21% until day 8 [21]. IL15 is crucial for NK cell survival, proliferation, and effector function. However, the first-in-human trials using systemic rhIL15 and IL15 super agonist complexes in patients treated with haploidentical NK cell therapy for advanced AML, showed expansion of host CD8^+^ T cells which resulted in CRS and rejection of allogeneic donor NK cells [10, 37]. Alternatively, multiple trials have combined NK cell administration with sc IL2 support, with limited described toxicity [3–5, 8, 9, 36]. Subcutaneous IL2 injections following high-dose chemotherapy and autologous SCT significantly expanded the number of circulating NK cells in vivo [38]. Miller and colleagues showed that there is a correlation between in vivo NK cell persistence and expansion of ≥ 100 donor NK cells/µl blood at day 7–14 after infusion in combination with in vivo IL2 boosting and achievement of CR in relapsed/refractory AML patients [5]. Our RNK001 cells show expression of CD25, the high affinity IL-2R, which may be advantageous when using low dose IL2. In our study, RNK001 cells will be infused without IL2, with low dose IL2 (3.0 × 10^6^ units/dose) or high dose IL2 (6.0 × 10^6^ units/dose). Subsequently both toxicity and efficacy of these treatment regimens can be compared.

To broaden the applicability of NK cell immunotherapy further improvements to the RNK001 product can be warranted. Currently, personalized RNK001 products are produced in 5 weeks. Including lead times this may result in exclusion of patients due to disease progression or deteriorating condition. Therefore, ongoing pre-clinical development is focused on generating a cryopreserved off-the-shelf product. The limited need for HLA matching allows for great flexibility in donor choice which makes banking a variety of common haplotypes and pooling of cryopreserved RNK001 cells highly feasible. Having a bank of cryopreserved RNK001 products available will greatly reduce time to infusion, allow for multiple infusions and may greatly reduce production cost. Other important ongoing pre-clinical developments to the RNK001 product include genetic modifications to improve tumor specific targeting and in vivo persistence. Liu et al. administered CAR-transduced UCB-derived-NK cells in CD19-positive lymphoid tumor patients that received Cy/Flu followed by a single infusion of CAR-NK cells at escalating doses. The NK cells not only expressed anti-CD19 CAR, but also IL15. The infused CAR/IL15-NK cells expanded and persisted at low levels for at least 12 months [39]. No adverse events such as GvHD, CRS or neurotoxicity were seen and a majority of the patients responded. Our platform is attractive for genetic engineering as well. Promising targets for CAR-NK cell therapy in AML patients include CD123 or CLL1 (CLEC12A) in combination with IL15/IL15Rα to improve persistence and expansion locally.

Altogether, this study shows the development of a GMP-compliant IL15/IL12/SR1-based HSPC-NK cell platform to generate large numbers of highly active NK cells. This method was validated and is now actively used in ongoing clinical trials. Meanwhile, future developments aim at optimization of the RNK001 product using genetic engineering and off-the-shelf availability. This platform will broaden the application of NK cell immunotherapy for AML/MDS-EB2 and potentially other malignancies.

## Supplementary Information

Below is the link to the electronic supplementary material.

Supplementary file1 (DOCX 657 kb)

## Data Availability

The datasets generated and analyzed during the current study are available from the corresponding author on reasonable request.
