# Supplementary material for: Good manufacturing practice production of CD34+ progenitor-derived NK cells for adoptive immunotherapy in acute myeloid leukemia
Source: Cancer Immunol Immunother. 2023 Jul 21;72(10):3323–35. doi: 10.1007/s00262-023-03492-6 (PMC10491545; doi:10.1007/s00262-023-03492-6)
Supplement: Supplementary file 1 — Supplementary file1 (DOCX 657 kb) [file 262_2023_3492_MOESM1_ESM.docx]

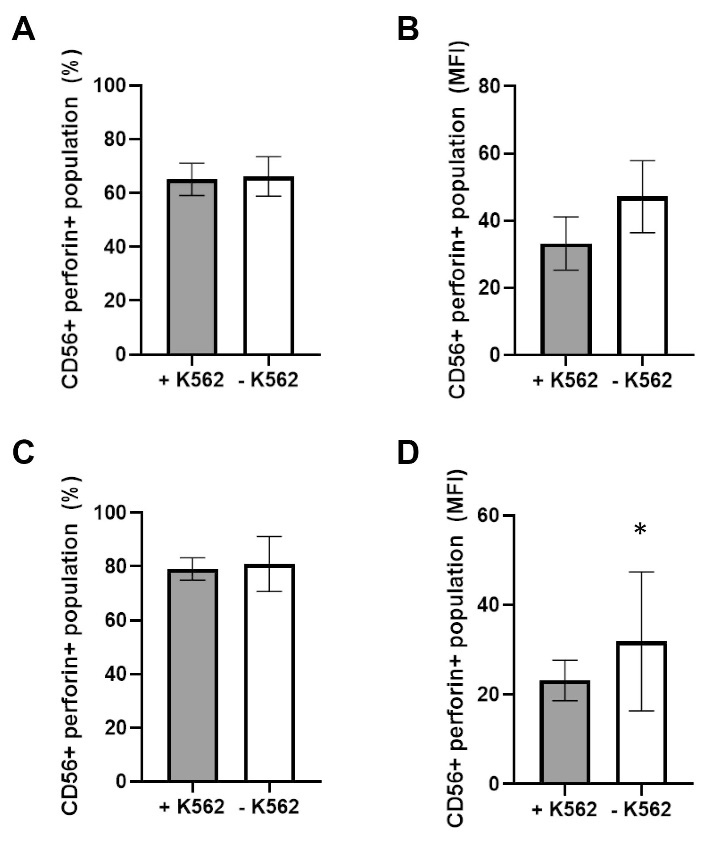


**Supplemental Figure 1 Dynamics in CD56^+^perforin^+^ population after stimulation with K562.** Percentage (A) and MFI (B) of CD56^+^perforin^+^ NK cells, depicted in figure 1I, with or without 4h stimulation with K562 E:T ratio 1.5:1 (n=3, measured in singlet (unstimulated, mean ± SD)) or duplo (stimulated (mean ± SEM) ns. Percentage (C) and MFI (D) of CD56^+^perforin^+^ NK cells, depicted in figure 2F, with or without 4h stimulation with K562 E:T ratio 1.5:1 (n=6, measured in singlet (unstimulated, mean ± SD)) or duplo (stimulated (mean ± SEM) p = 0.03. Statistical analyses performed with Wilcoxon matched-pairs signed rank test.


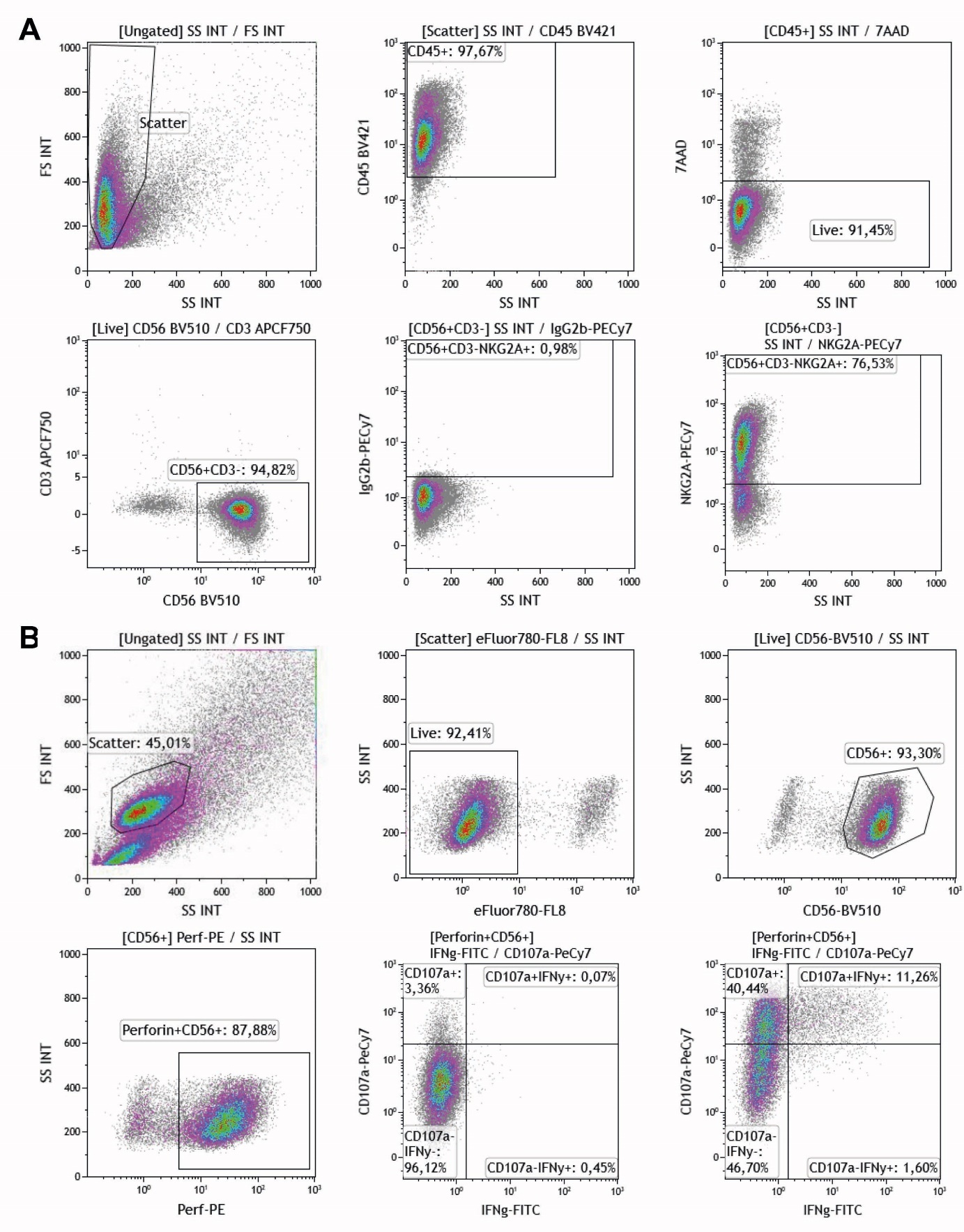


**Supplemental Figure 2 Gating strategy.** Example flowcytometry plots and gating strategy used to determine reported marker expression. (A) For surface markers, debris is gated out using a lymphocyte gate (Scatter). Next, cells are gated for CD45 followed by gating Live cells. NK cells are then gated based on CD56+ and CD3-. Receptor expression gates (in this example NKG2A) were determined based on IgG isotype controls which were set as close to 1% as possible as shown side by side. (B) For intracellular markers, debris is gated out using a lymphocyte gate (Scatter). Next, live cells are gated followed by gating NK cells based on CD56+. Cells are then gated on Perforin+. This population is then used to determine CD107a+ and IFNy+ after stimulation with K562 target cells. Gates are based on the unstimulated control as shown side by side.

|  | Final product composition | | | | | |
| --- | --- | --- | --- | --- | --- | --- |
|  | *CD56+ (%)* | *CD34+ (%)* | *CD3+(%)* | *CD19+(%)* | *CD14+ (%)* | *CD15+(%)* |
| NK1 | 75 | 1.6 | <0.1 | <0.1 | ND* | ND* |
| NK2 | 92 | 0.1 | <0.1 | <0.1 | ND* | ND* |
| NK3 | 96 | 0.1 | <0.1 | <0.1 | ND* | ND* |
| NK4 | 88 | 0.1 | <0.1 | <0.1 | 8 | 5 |
| NK5 | 73 | 0.2 | <0.1 | <0.1 | 25 | 16 |
| NK6 | 70 | 0.1 | <0.1 | <0.1 | 3 | 4 |
| NK7 | 92 | 0.0 | <0.1 | <0.1 | 2 | 4 |

*Not determined

**Supplementary table 1 Final product composition.**
